# Knowledge, Attitudes and Practices toward Energy Drinks among Adolescents in Saudi Arabia

**DOI:** 10.5539/gjhs.v6n2p42

**Published:** 2013-11-27

**Authors:** Abdulrahman O. Musaiger, Nisreen Zagzoog

**Affiliations:** 1 Arab Center for Nutrition, Bahrain; 2 Perfect Diet Center, Jeddah, Saudi Arabia

**Keywords:** adolescents, caffeinated drinks, energy drinks, Saudi Arabia

## Abstract

The objective of this study is to explore the knowledge, attitudes and intake of energy drinks among adolescents in Saudi Arabia. A multi-stage stratified sampling procedure was carried out to select 1061 school children aged 12–19 years, from Jeddah city, Saudi Arabia. A short self-reported questionnaire was administrated in order to collect the data. Of adolescents in the study, 45% drank energy drinks (71.3% males and 35.9% females; P<0.001). Advertisements were the main source of information on energy drinks (43%). The major reasons for consuming energy drinks were taste and flavour (58%), to ‘try them’ (51.9%) and ‘to get energy’ (43%), albeit with significant differences between genders (P<0.001). About half of the adolescents did not know the ingredients of these drinks, and 49% did not know that they contain caffeine (P-values <0.006 and <0.001 between genders, respectively). The greater majority (67%) considered energy drinks to be soft drinks. The study indicates the need for Saudi adolescents to be warned on the over-consumption of energy drinks. The study brings to attention the need for educational programmes related to increasing awareness in the community of the health effects related to high consumption of energy drinks.

## 1. Introduction

The consumption of energy drinks has become a popular practice worldwide, especially among younger populations. The extensive advertising of these drinks and their accessibility in grocery stores, convenience stores and supermarkets has made them both acceptable and readily available for all age groups in a population (Babu et al., 2008). It was reported that energy drinks are available to buy in more than 140 countries, and half of the consumers of these drinks consisted of children, adolescents and young adults (Seifert et al., 2011). It is claimed in the marketing of these energy drinks that they provide immediate energy, decreased fatigue, and improved performance (Reissig et al., 2009). These claims may encourage consumers to increase their intake of energy drinks. In Germany, for example, energy drinks are consumed by 30–50% of adolescents and young adults (Viell et al., 1996); about 51% of college students in the USA drink energy drinks more than once a month (Malinauskas et al., 2007); and in the United Arab Emirates (UAE), 92% of college students have consumed energy drinks (Jacob et al., 2013).

The main active ingredients in most energy drinks are caffeine, amino acids, B-vitamins, and herbal supplements (Gunja & Brown, 2012). Frequent consumption of energy drinks was positively associated with several behavioural problems, such as substance abuse, fighting, failure to wear a seatbelt, smoking and drinking alcohol (Miller, 2008). Although the caffeine level in energy drinks is safe for many consumers when used in moderation (caffeine intake of less than 300 mg/day), high caffeine content poses serious health risks for certain people, such as pregnant and lactating women, and patients with diabetes, peptic ulcers, hypertension, congestive heart failure and dysrhythmias (Clauson et al., 2008).

Athletes were initially the primary target consumers of energy drinks companies. However, as the energy drinks market rapidly grew and expanded, athletes were no longer the main target. Today, energy drinks manufacturers target teenagers and young adults (18–34 year olds) (Heckman et al., 2010). For example, 34% of 18–24 year olds in the USA are regular energy drinks consumers (O’Brien et al., 2008). In Italy, Gallimberti et al. (2013) reported that the consumption of energy drinks increased significantly with age of adolescents, from 17.8% among sixth grade to 56.2% among eight grade students. There are few studies into the consumption of energy drinks, and most of these concentrate on college students. Adolescents have received less attention than other age groups in this regard, although they are the main victims of the side-effects of high consumption of energy drinks (Pennington et al., 2010). To our knowledge, there has been no research related to the consumption of energy drinks among school children in Saudi Arabia, and potentially none in any Arab countries. Given the lack of studies on energy drinks consumption, the rapid growth of these drinks in Saudi market and the negative health effects of energy drinks, the current researchers carried out this study to explore the intake and attitudes of Saudi adolescents towards energy drinks.

## 2. Methods

The target group for this study was schoolchildren aged between 12 and 19 years of age. A multi-stage stratified sampling procedure was used to select the children from schools in Jeddah city, Saudi Arabia. Eight public and four private preparatory and secondary schools were first selected by a simple random method from the list of schools in Jeddah city. A single class from each educational level (levels 8 to 12) was then selected from each school by a simple random method. The total sample was 1061 (562 males and 499 females). Ethical approval for the study was obtained from the School Health Directorate, Ministry of Education, Jeddah city, Saudi Arabia.

A short questionnaire extracted from a previously validated questionnaire was relied upon (after some modifications to include energy drinks) (Musaiger et al., 2011).The questionnaire consisted of questions on the frequency of energy drink intake per week, as well as knowledge and attitudes toward these drinks. The questionnaires were distributed to the students in their classes and explained to them by a qualified nutritionist. The data were analysed using the *Epi Info statistical software package* (CDC, 2012). A chi-squared test was used to determine the significance of the association between energy drinks practices and gender.

## 3. Results

The frequency of Saudi adolescents’energy drink intake per week, their source of information and reasons for intake are presented in Table 1. Of these adolescents, 31.9% of males and 24.7% of females drank energy drinks 1–2 days per week, with a significant difference between the genders (P<0.001). Advertisements were the main single source of information on energy drinks (51.6% and 33.9% in males and females, respectively). The main reasons for consuming energy drinks were for their taste and flavour (58.4%), in order to ‘try them’ (51.8%) and to ‘get energy’ (43%), with significant differences between males and females (P<0.001).

**Table 1:** Intake of energy drinks, source of information and reasons for intake by Saudi adolescents

Intake and Reasons	Male (n=562)		Female (n= 499)		*P*-value
	No.	%	No.	%	
**Intake of energy drinks (cans per week)**					
Do not intake	161	28.7	320	64.1	<0.001
1–2	179	31.9	123	24.7	
3–4	97	17.2	36	7.2	
5+	125	22.2	20	4.0	
**Source of information on energy on energy drinks**					
Advertisements	290	51.6	169	33.9	<0.001
Peers	124	22.1	43	8.6	
Family members	19	3.4	45	9.0	
Several sources	129	22.9	242	48.5	
**Reasons for intake of energy drinks^1^**					
Taste and flavour	370	65.8	250	50.1	<0.001
Provide energy to body	267	47.5	189	37.9	
Build the body	101	18.0	35	7.0	
Follow/Imitate the peer	56	10.0	99	19.8	
Sample/Trial	258	45.9	292	58.5	

1 Values do not add up to 100%, because of multiple answers.

Knowledge and attitudes toward energy drinks among Saudi adolescents are given in Table 2. The majority of adolescents (54.3% males; 67.7% females) believed that the price of energy drinks is reasonable. About half of adolescents (47% male; 52.3% female) did not know the ingredients of energy drinks (P<0.006), and only 53.2% of males and 48.3% of females knew that these drinks contained caffeine (P<0.001). However, only 27.2% of males and 5.8% of females knew that energy drinks contain vitamins (P<0.001). The great majority of adolescents (67%) viewed energy drinks as soft drinks.

**Table 2:** Knowledge and attitudes towards energy drinks among Saudi adolescents

Knowledge and Attitudes	Male (n=562)		Female (n= 499)		*P*-value
	No.	%	No.	%
**Do you think the price of energy drinks is reasonable?**					
Yes	305	54.3	338	67.7	<0.001
No	257	45.7	161	32.3	
**What are the ingredients of energy drinks?**
Do not know	264	47.0	261	52.3	<0.006
Elements to boost energy	91	16.2	45	9.0	
Stimulants	57	10.1	53	10.6	
Both	150	26.7	140	52.3	
**Do you think that energy drinks contain caffeine?**					
Do not know	212	37.7	241	48.3	<0.001
Yes	299	53.2	241	48.3	
No	51	9.1	17	3.4	
**Do you think that energy drinks contain vitamins?**					
Do not know	202	36.0	177	35.5	<0.001
Yes	153	27.2	29	5.8	
No	207	36.8	293	58.7	
**Do you consider energy drinks to be the same as soft drinks?**					
Yes	386	68.7	322	64.5	<0.152
No	176	31.3	177	35.5	

## 4. Discussion

This study indicates an alarming situation concerning energy drinks intake among adolescents in Saudi Arabia. About 55% of adolescents consumed energy drinks once or more each week, and 43% believed that these drinks provide immediate energy. Furthermore, about half of adolescents had no knowledge of the ingredients in energy drinks, and a similar amount did not know that these drinks contain caffeine. Even more worrisome was that most of the adolescents considered energy drinks to be soft drinks.

Energy drink consumption has grown very rapidly since they were first introduced in Saudi Arabia at the beginning of 2000; and with a society consisting of more than 50% children and young adults; it is more than likely that this growth will continue. It has been estimated that the energy drinks market will double its income in Saudi Arabia between 2012 and 2016 (Business Monitor International, 2012). The frequency of energy drinks consumption by Saudi adolescents is relatively high, especially among males, with 22% of males consuming five cans or more each week. This percentage is higher than that reported among young adults in Western countries (Viell et al., 1996; Malinauskas et al., 2007). A previous study on Saudi adolescents aged 14-19 years reported that 16.3% of males and 8.5% of females consumed energy drinks more than three days per a week (Al-Hazzaa et al., 2011). These proportions are lower than that found in this study. Male adolescents reported higher rates of consumption than females. This finding conflicts with that of college students in the USA, where females are the predominant consumers (Malinauskas et al., 2007), but on agreement with studies on adolescents at same age group in several Arab Gulf countries (Al-Hazzaa et al., 2012; Allafi et al., 2013; Kilani et al., 2013). The high consumption of energy drinks by males in this study may be attributed to that male Saudi adolescents are more likely to practice sport than their female counterparts, and consequently they consume these drinks to provide them energy for practicing sport and to build their bodies (Table 1).

Advertisements were the main single source of information on energy drinks among Saudi adolescents (43%). Energy drinks are promoted for their health benefits, as well as for increasing attention, endurance and performance, providing energy and aiding weight loss. Therefore, consumers may falsely believe that the more they consume the better (Reissig et al., 2009). About 43% of Saudi adolescents believed that energy drinks provide quick energy; conversely, it was found that 65% of university students in the USA consumed energy drinks to increase energy (Malinauskas et al., 2007). There is insufficient evidence to conclude that energy drinks are more effective at increasing energy than traditional caffeinated drinks such as coffee, tea and cola (Clauson et al., 2008). None of our studied Saudi adolescents mentioned that energy drinks improved their cognitive function. A study in the UAE reported that 85% of college students believed that energy drinks would enhance their brain development (Jacob et al., 2013). Therefore, education on the health aspects of energy drinks should be provided to the public through various mass media. Censorship of the unscientific claims in advertisements may participate in reducing the misconception about energy drinks among both the adolescents and adults.

A lack of knowledge among adolescents on the active ingredients in energy drinks is highly reported in this study, with 49% of these adolescents reporting that they did not know that energy drinks contained caffeine. Due to lack of data on adolescents regarding this finding, we compared such result with university students in UAE as the socio-cultural background is very close. In the UAE, 95% of university students had no information on the high caffeine content of energy drinks (Jacob et al., 2013). Adolescents who had a high consumption of these drinks reported specific side-effects, including jitteriness, nervousness, dizziness, inability to focus, difficulties with concentration, gastrointestinal upset and insomnia (Pennington et al., 2010). Several different brands of energy drinks are available in the market, with caffeine level ranging from moderate (50 mgper can or bottle) to alarming levels (505 mg) (Reissig et al., 2009). In spite of this, the caffeine content (mg/ml) of energy drinks is close to that of many coffee beverages, and energy drinks are available in higher volume packages, with the result of higher intakes of caffeine (Babu et al., 2008). However, half of adolescents in this study knew that energy drinks contain caffeine, and in spite of that they consume these drinks. This is mainly due to the fact that these adolescents do not aware about the side effects of consuming high dose of caffeine. In USA, Ward (2009) found that although the adolescents had negative attitudes toward caffeine, they continued to intake energy drinks.

There are two limitations of this study that should be mentioned. First, as in any self-reported research there is a drawback of social desirability. Second, the sample included adolescents in one city (Jeddah), and therefore it is not necessarily representative of adolescents in Saudi Arabia.

## 5. Conclusion

In conclusion, the current study showed that a high proportion of Saudi adolescents consumed a relatively high amount of energy drinks; furthermore, they were likely to be unaware of the side-effects on their health, especially with no warning on the products. It was recommended that any labelling and marketing of energy drinks should include appropriate health warnings. In USA, it was found that having seen warning labels on energy drinks can significantly reduce the odds that the youths would consume energy drinks (Ward, 2009) However, such finding need further investigation. Also, as with soft drinks, it is important that caffeine content in energy drinks is regulated (Gunja & Brown, 2012). Health education programmes either in mass media or in schools should be established so as to educate the public on the health aspects of energy drinks. Further in-depth studies on the energy drinks consumption behaviour among adolescents and youth in the Arab world are at most needed. We hope that this study provides *base-line data* to carry out such in-depth studies.
